# Virus-Driven Carcinogenesis

**DOI:** 10.3390/cancers13112625

**Published:** 2021-05-27

**Authors:** Yuichiro Hatano, Takayasu Ideta, Akihiro Hirata, Kayoko Hatano, Hiroyuki Tomita, Hideshi Okada, Masahito Shimizu, Takuji Tanaka, Akira Hara

**Affiliations:** 1 Department of Tumor Pathology, Gifu University Graduate School of Medicine, Gifu 501-1194, Japan; h_tomita@gifu-u.ac.jp (H.T.); ahara@gifu-u.ac.jp (A.H.); 2 Department of Gastroenterology, Gifu University Graduate School of Medicine, Gifu 501-1194, Japan; idetat@gifu-u.ac.jp (T.I.); shimim@gifu-u.ac.jp (M.S.); 3 Department of Laboratory Medicine, Gifu University Graduate School of Medicine, Gifu 501-1194, Japan; 4 Laboratory of Veterinary Pathology, Joint Department of Veterinary Medicine, Faculty of Applied Biological Sciences, Gifu University, Gifu 501-1194, Japan; akatsuki@gifu-u.ac.jp; 5 Department of Obstetrics and Gynecology, Gifu University Hospital, Gifu 501-1194, Japan; htnhtn@gifu-u.ac.jp; 6 Department of Emergency and Disaster Medicine, Gifu University Graduate School of Medicine, Gifu 501-1194, Japan; hideshi@gifu-u.ac.jp; 7 Department of Diagnostic Pathology (DDP) and Research Center of Diagnostic Pathology (RC-DiP), Gifu Municipal Hospital, Gifu 500-8513, Japan; takutt@gmhosp.gifu.gifu.jp

**Keywords:** tumor virus, HPV, HBV, HCV, EBV, KSHV, MCV, HTLV-1, HIV-1

## Abstract

**Simple Summary:**

Carcinogens, causes of cancer, are usually invisible and therefore in vivo carcinogenesis is difficult to detect. Tumor viruses, definitive carcinogens, are also usually unremarkable, particularly due to latent infection. However, recent developments in tumor virology are unraveling how a single infected cell becomes a life-threatening cell population from a molecular perspective. The recognition and characterization of virus-driven carcinogenesis is the first step in the eradication of tumor virus-associated cancer.

**Abstract:**

Cancer arises from the accumulation of genetic and epigenetic alterations. Even in the era of precision oncology, carcinogens contributing to neoplastic process are still an important focus of research. Comprehensive genomic analyses have revealed various combinations of base substitutions, referred to as the mutational signatures, in cancer. Each mutational signature is believed to arise from specific DNA damage and repair processes, including carcinogens. However, as a type of carcinogen, tumor viruses increase the cancer risk by alternative mechanisms, including insertional mutagenesis, viral oncogenes, and immunosuppression. In this review, we summarize virus-driven carcinogenesis to provide a framework for the control of malignant cell proliferation. We first provide a brief overview of oncogenic viruses and describe their implication in virus-related tumors. Next, we describe tumor viruses (HPV, Human papilloma virus; HBV, Hepatitis B virus; HCV, Hepatitis C virus; EBV, Epstein–Barr virus; Kaposi sarcoma herpesvirus; MCV, Merkel cell polyoma virus; HTLV-1, Human T-cell lymphotropic virus, type-1) and tumor virus-related cancers. Lastly, we introduce emerging tumor virus candidates, human cytomegalovirus (CMV), human herpesvirus-6 (HHV-6) and adeno-associated virus-2 (AAV-2). We expect this review to be a hub in a complex network of data for virus-associated carcinogenesis.

## 1. Introduction

Cancer is classified as a cell proliferative lesion according to its histology and primary site. However, cancer frequently shows variation among individuals and unique histological features, and no single histological type is identical to the others. Consistent with this notion, next-generation sequencing-based genomic analyses have shown that each histological cancer type shows intertumoral heterogeneity owing to genomic and epigenomic alterations in cancer-related genes. Such comprehensive analyses clarify not only the status of established cancer-related genes but also collateral factors, including tumor mutational burden and microsatellite instability. These additional factors are closely associated with past DNA damage and repair responses in cancer and play an important role in deciding optimal molecular therapies. These comprehensive analyses allow us to understand individual cancer in a chronological framework and, thus, the molecular classification of cancer is an emerging area in research and healthcare [1].

Will the emerging genomic classification really be the best and only approach in clinical oncology? Another approach for the classification of cancer is to investigate possible predisposing factors. These factors can be hereditary elements [2] and environmental factors, called carcinogens. Among carcinogens, oncogenic viruses predispose carriers to specific types of cancer in infected cells. Of note, studies of virus-driven carcinogenesis have generated insight into oncogenes [3] and thereby to carcinogenesis. Oncogenic viruses are a promising target for cancer prevention, diagnosis, and therapy.

In this review, we summarize virus-driven carcinogenesis to understand the malignant cell proliferation as controllable. We initially provide brief remarks on human viruses and oncogenic viruses. Next, we provide a detailed overview of every established virus-driven carcinogenesis. Lastly, we introduce miscellaneous infectious agents, including emerging tumor virus candidates.

## 2. Overview of Tumor Viruses

Inorganic particles that are infectious to humans, called human viruses, were discovered at beginning of the 20th century [4]. Despite possessing genetic information like other organisms, viruses fail to self-replicate. Viral replication requires an environmental factor, i.e., an appropriate host cell. Occasionally, virus activity leads to the illness and death of the host, resembling many ecological interactions. Owing to extensive research, the current virus taxonomy is highly complex and is beyond the scope of this review [5]; accordingly, we focus on the relationships between cancer and tumor viruses.

Tumor viruses, also called oncogenic viruses or oncoviruses, are associated with one-ninth of cancer cases worldwide [6,7,8,9]. As summarized in Table 1, principal oncogenic viruses can be classified into six families. The International Agency for Research on Cancer (IARC) recognizes several tumor viruses as human carcinogens [10,11]. Hepatitis B virus (HBV), hepatitis C virus (HCV), Epstein–Barr virus (EBV), Kaposi sarcoma herpesvirus (KSHV), human papilloma viruses (HPVs), especially type 16, and human T-cell lymphotropic virus, type-1 (HTLV-1) are classified as “carcinogenic to humans“ (Group 1) [10]. Polyoma viruses were later added to the IARC monograph series. Merkel cell polyoma virus (MCV) is classified as “probably carcinogenic to humans” (Group 2A); BK virus and JC virus are classified as “possibly carcinogenic to humans” (Group 2B); SV40 virus is regarded as “not classifiable as to its carcinogenicity to humans”(Group 3) [11]. Recently, evidence for the carcinogenicity of MCV has accumulated [12]. 

Evidence for virus-driven carcinogenesis is summarized as follows [15]: the existence of viral DNA in tumor tissues, transformation by viral genes in model systems, generation of a neoplastic phenotype in response to the persistent expression of a vital oncogene or modification of host genes, and epidemiological data indicating that viral infections represent a major risk for carcinogenesis.

Although tumor viruses possess several “hallmarks of cancer” [16,17,18], there is a long lag between virus-driven carcinogenesis and the initial infection [19]. This slow neoplastic process can explain why the virus benefits from self-propagation, rather than host death. 

Direct carcinogenicity of tumor viruses is due to insertional mutagenesis and viral oncogenes. Virus integration, distinct from viral contamination, is a cause of carcinogenesis [20]. Insertional mutagenesis refers to the alteration of gene structure or transcript levels by sequential alterations resulting from viral DNA integration. It is applicable to the constitutive expression of not only host but also viral genes. In contrast, expression of viral oncogenes acts as cancer driver genes in infected cells.

Indirect carcinogenicity of tumor viruses involves chronic inflammation and an immunosuppressive status arising from the infected cells. Chronic inflammation leads to DNA damage accumulation in tissue stem cells by repeated tissue injury and regeneration [21,22]. Some virus-associated cancers harbor specific DNA damage patterns, also called the mutational signatures [23,24].

Immunosuppression/immunodeficiency is a risk factor for cancer, including virus-driven carcinogenesis [25]. Interestingly, most tumor viruses also attenuate the host immune state [18]. Consistent with this observation, the IARC classified human immunodeficiency virus, type 1 (HIV-1) as a Group 1 agent, indirectly associated with the cancer risk via immunosuppression [10]. A decrease in CD4+ T-cells by HIV-1 infection leads to acquired immunodeficiency syndrome (AIDS) [26]. Despite rapid progress in therapeutic strategies for HIV-1 [27], there is no established treatment to eliminate the retrovirus. In this clinical context, HIV-1 carriers and patients with AIDS develop other tumor virus-associated lesions [28].

Recently, the Pan-Cancer Analysis of Whole Genomes (PCAWG) Consortium reanalyzed the whole-genome sequencing data for 2656 cancer cases involving 38 tumor types [29]. Although clear evidence for the “hit-and-run theory” in human cancer has not been obtained [30], tumor viruses in cancer are detectable by massive DNA sequencing [31,32]. Consistent with the well-established etiologies, EBV is detected principally in head and neck, gastric, and esophageal cancers. HBV is associated with liver cancer. Alpha-papilloma viruses are found in head and neck and uterine cervical cancers, except for cases of non-specific viral contamination. In addition to tumor virus-associated events, transcripts from a human endogenous retrovirus, *ERV1*, are abundant in chronic lymphocytic leukemia and clear cell renal cell carcinoma [33].

## 3. Diversity of Tumor Viruses

In this section, we describe the details of virus-driven carcinogenesis according to viruses and their target organs (Table 2).

### 3.1. Human Papilloma Virus (HPV)

Dermatotropic HPV was first isolated from a skin wart in 1973 [34] and a mucosotropic type, also called type 16, was subsequently identified from a cervical cancer sample by the research group of Dr. Zur Hausen [35]. At the beginning of the 21st century, more than 100 types of papilloma virus were identified [36]. HPVs, including alpha- and beta-papillomaviruses, principally infects the squamous epithelium of the human mucosa and skin. Persistent HPV infection leads to squamous proliferative lesions, also called verruca/wart, papilloma, and condyloma [37,38]. 

Occasionally, HPV-related lesions give rise to malignant tumors due to the unique genomic characteristics of the high-risk HPV type. According to the IARC monograph, high-risk HPVs, which belong to alpha-papillomavirus, are types 16, 31, 52, 58 (clade A9); 18, 45, 59 (clade A7); 51 (clade A5); 56, 66 (clade A6) [39]. Of these HPVs, types 16 and 18 are the main causes of cervical cancer via the activation of oncogenes E6 and E7. In brief, E6 induces ubiquitination-mediated p53 degradation and, thus, blocks the p53 pathway [40,41,42]. In contrast, E7 inactivates the Rb pathway by calpain-mediated proteasomal degradation [43,44]. In addition, these oncoproteins function as immunosuppressors by the blockade of interferon pathways [45,46,47,48], indicating that the immunomodulatory effects promote the persistent infection of high-risk HPVs that initiates neoplastic change.

The integration of HPV into the human genome is an essential step in cervical carcinogenesis. This event leads to not only insertional mutagenesis but also to the persistent expression of the viral oncoproteins E6 and E7 [49]. HPV integration frequently occurs at fragile sites [50]. A recent genomic analysis revealed that the integration sites contain microhomologous sequences [51], a cause of microhomology-mediated break-induced replication [52].

Although HPV-associated cancer occurs in various body parts, including the oral cavity [53], anus [54], vagina [55], vulva [56], and penis [57], we focus only on two major cancer types, cancers of the uterine cervix [58] and head and neck [59].

A prototypical HPV-related carcinoma, cervical cancer had the fourth highest incidence and mortality rate among female cancers in 2020 [60]. Cervical cancer is almost always associated with high-risk HPVs and approximately a quarter of cases harbor an *PIK3CA* mutation, especially E542K and E545K [61]. These mutations link the APOBEC family gene-related mutational signature with HPV-related carcinogenesis [62,63,64]. 

Based on accumulating evidence, the most recent WHO classification regards HPV-independent cervical cancer as a distinctive diagnostic entity [65]. Interestingly, these HPV-negative categories include not only squamous lesions but also glandular lesions [66,67] and neuroendocrine carcinoma [68]. These entities are considered an unfavorable prognostic group [69] and alternative forms of carcinogenesis. Therefore, the dual model of cervical carcinoma is reasonable for clinical practice and translational research. To determine whether cervical carcinoma is associated with high-risk HPV, the surrogate marker p16 is used in practice [70]. The overexpression of p16 arises from Rb inactivation by the viral oncogene E7 [71]. To eliminate HPV-positive cervical cancer, the main strategy consists of three preventive tactics: HPV vaccination, screening, and surgical treatment for precancerous lesions [72]. 

Similar to genital lesions, an association between HPV and laryngeal papilloma was reported in the 1980s [73]. A small subset of cases of head and neck cancer, especially oropharyngeal tumors, is also associated with HPV [74,75]. Consistent with cervical cancer, p16 positivity is a diagnostic marker for HPV-associated squamous cell carcinoma. Histologically, HPV-related head and neck cancer is a non-keratinizing type squamous cell carcinoma [76]. A recent TCGA comprehensive analysis [75] revealed that HPV-positive head and neck squamous cell carcinomas (HPV+HNSCC) are distinct from HPV-negative squamous cell carcinomas (HPV-HNSCC), which are probably related to the other carcinogens, including alcohol and tobacco [77]. HPV+HNSCC sometimes shows deletions or truncating mutations in *TRAF3,* involved in anti-viral responses [78]. HPV+HNSCC harbors a low frequency of *TP53* mutations and is associated with a relatively favorable prognosis comparing with that of *TP53*-mutated and/or *CCND1*-amplified HPV-HNSCC. HPV+HNSCC harbors rare alterations of *CDKN2*, which encodes p16, whereas HPV-HNSCC commonly lacks the 9p21.3 region, including *CDKN2*. Consistent with findings for cervical cancer, the genomic integration of HPV is randomly detected in 80% of HPV+HNSCC.

In contrast, the frequency of *TP53* mutations in HPV-HNSCC is high (84%) [75]. *TP53* mutations in HPV-negative squamous cell carcinoma are reproducible events in other organs [79,80,81,82]. In addition, aberrant p53 expression has been reported in several premalignant lesions [83,84,85,86]. These observations suggest that the dysregulation of p53 is an essential and/or early step in squamous cell carcinogenesis.

### 3.2. Hepatitis Virus

Liver cancer ranks sixth and fourth in incidence and mortality, respectively, in the world, with an estimated 905,677 new cases and 830,180 deaths in 2020 [60]. The most common cancer type of liver cancer is hepatocellular carcinoma (HCC), which arises from a well-established cause in most cases [87]. HCC is associated with chronic liver disease, including persistent hepatitis B virus (HBV) and/or hepatitis C virus (HCV) infection [88,89]. In the following sections, we describe the features of each hepatitis virus.

#### 3.2.1. Hepatitis B Virus (HBV)

The discovery of Australia antigen, now known as HBs, in 1964 initiated HBV research [90]. The subsequent discovery of HBV particles by Dane in 1970 [91] was followed by the cloning of HBV [92]. HBV is subclassified into nine genotypes [93], which are associated with clinical and epidemiological features [94]. This genetic variation is probably due to reverse transcription in viral replication [95]. Similarly, the genomic integration of HBV into host DNA frequently occurs, indicating that insertional mutagenesis is a major oncogenic event in HBV-related carcinogenesis [96]. The HBV integration breakpoints are random, except for several hotspots [97]. Rapid progress has been made in preventive and therapeutic interventions aimed at suppressing HBV infection and hepatitis [98,99,100]. 

HCC is a heterogeneous malignancy with respect to both morphological and genetical features [101]. A recent genomic analysis revealed the features of HCC [97,102,103,104]. The common driver events in HCC are the dysregulation of p53, TERT, and WNT pathways, principally due to mutations in *TP53*, the *TERT* promotor, and *CTNNB1,* respectively [105,106,107]. Consistent with the features of HBV, the majority of HCC cases show genomic integration of HBV [97]. Compared with the other etiology, HBV-related HCC frequently involves insertional mutagenesis, including mutations in *KMT2B* [108,109], *KMT2D* [104], *CCND1* [104], *CCNE1* [104], and *TERT*. Indeed, one-third of TERT dysregulation events arise from HBV integration [102]. In contrast, there is no clear evidence for HBV integration-associated copy number amplification [102]. In addition, the frequency of *TP53* mutations in HBV-related HCC is high [103]. A recent TCGA analysis suggested that there is an association between HBV infection and exposure to Aflatoxin A, a liver carcinogen [104].

HBV is also a risk factor for small duct type intrahepatic carcinoma, which shows a biliary phenotype [110,111]. Although the origin of this cancer is considered liver progenitor cells [112] or transdifferentiated mature hepatocytes [113], the premalignant lesion remains unclear. Unlike HCC, this cancer typically express biliary markers EMA, CK7, and CK19, and lacks hepatocytic markers, including HepPar-1 and AFP. Unique genomic features of small duct type intrahepatic carcinoma are *IDH1/2* [114] and *BRAF* mutations and *FGFR2* translocation [111]. In addition, HBV-associated intrahepatic adenocarcinoma also harbors frequent *TP53* mutations [115].

#### 3.2.2. Hepatitis C Virus (HCV)

In 1989, the genetic sequence derived from HCV was first isolated as a transfusion-mediated hepatitis virus, unrelated to hepatitis A virus or HBV [116]. The majority of non-A, non-B hepatitis is due to HCV [117]. These research milestones have contributed the reduction of transfusion-mediated hepatitis. The sequence of the HCV genome was then completed by the two groups [118,119], and HCV-induced hepatitis was finally demonstrated by an in vivo animal experiment [120]. Further evolution of HCV experimental models [121,122] has resulted in the development of novel therapeutic agents. A combination of direct acting antivirals (DAAs) has replaced standard interferon-based treatment [123]. 

Unlike HBV, HCV clearly induces hepatitis [124]. In addition to indirect effects [125], several HCV proteins have oncogenic properties [126,127,128]. Therefore, HCV eradiation by DAAs suppresses hepatocellular carcinogenesis [129,130]. However, liver cirrhosis confers a cancer risk after the elimination of the virus.

Although both HBV and HCV predispose patients to liver malignant neoplasia via chronic liver damage, HBV-associated HCC is believed to have high oncogenic potential. The frequencies of TERT promotor mutations and p16 silencing are higher for HCV-associated HCC than for HBV-associated and non-viral HCC [102]. In addition, HCV and HBV show distinct methylation patterns [104]. The virus infection status, for both HBV and HCV, is unrelated to the HCC immunophenotype, including checkpoint genes [104]. 

Epidemiological data suggest that HCV infection is associated with a predisposition towards small duct type intrahepatic cholangiocarcinoma [131] and malignant lymphoma [132]. Interestingly, these lymphomas sometimes regress after HCV elimination by antiviral agents [133,134]. A possible mechanism underlying lymphomagenesis is immune reactions between viral antigens and lymphocyte receptors.

### 3.3. Epstein–Barr Virus (EBV/HHV-4)

EBV, classified as a human herpesvirus 4 (HHV-4), was first isolated from Burkitt lymphoma (BL) by Epstein and Barr in 1964 [135]. A ubiquitous infectious agent, EBV initially infects B-cells or epithelial cells [136]. The infection pattens of EBV are divided in latent and lytic infection. In latent infection, EBV exists as an episome in the nuclei of infected cells. The latent infection is further classified into latency I–III [137], according to the expression patterns of latent genes (Table 3). 

To detect the latency pattern, immunostaining is used in clinical settings. EBNA2 expression is only found in latency III cells, whereas a lack of LMP1 indicates latency I cells. LMP1 induces the transformation of infected B-cells by activating CD40 signaling [138], whereas the transcription factor EBNA2 upregulates LMP1 expression [139,140]. Regardless of the latency pattern, EBV-encoded small RNA (EBER) [141,142,143] is consistently detected in EBV-infected cells [144]. Therefore, in situ hybridization for EBER is the most sensitive detection method for EBV-infected cells [145]. In the lytic infection, the sequential reactivation of lytic genes leads to the production of virus particles in order to propagate viral infection [146].

In 1958, Denis Burkitt reported a case series of pediatric small round cell tumors of the jaw [147]. BL is a truly B-cell malignancy whose histological pattern is described as a jigsaw puzzle or starry sky. The immunophenotype of BL shows the latency I pattern, indicating that the pathogenesis requires minimal latent proteins, EBER [148,149] and EBNA1 [150,151]. Rather, the driving force of BL is *c-myc* translocation, which is almost always detected in all three clinical types; endemic and sporadic BL and BL in immunocompromised hosts [152]. In addition, BL frequently has *TCF3* and *ID3* mutations [153,154,155,156]. BL is a highly aggressive tumor and is possibly curable by massive chemotherapy.

In addition to BL, various hematolymphoid neoplasms and/or proliferative disorders can be consolidated as EBV-associated diseases [152]. As shown in Table 4, EBV-associated hematolymphoid diseases consist of various diagnostic entities, including neoplasia of B-cell and NK/T-cell lineages, classical Hodgkin lymphoma, lymphoproliferative disorders, and infectious and reactive lesions. The clonality of EBV-associated lesions is demonstrable by the sequential analysis of EBV termini [157]. Notably, for each EBV-positive tumor, the EBV-negative counterpart and/or mimicry should be ruled out. For example, in a case of EBV-negative BL mimicry, Burkitt-like lymphoma has an 11q aberration without a *MYC* translocation [158,159]. EBV-associated lesions also include reactive lesions, probably due to EBER-mediated TLR3 signaling [160]. To understand the diversity of EBV-related hematolymphoid lesions, the precise classification based on the EBV infection status is essential.

Nasopharyngeal carcinoma, non-keratinizing type is associated with EBV, which is detected by in situ hybridization for EBER [216]. The typical histology, cancer cell nests intermingled with lymphocytes and plasma cells, is often called lymphoepithelial carcinoma [217]. Nasopharyngeal carcinoma typically shows latency II and, therefore, LMP-driven carcinogenesis [218,219]. In addition, EBV-positive nasopharyngeal carcinoma is accompanied by NF-κB activation [220]. Other characteristics of EBV-associated malignancies, e.g., nasopharyngeal carcinoma [221], Hodgkin’s lymphoma [222], and gastric cancer [223,224], include unique DNA methylation patterns, also called the CpG-island methylator phenotype (CIMP) [225]. These epigenetic changes are induced by DNMT1 upregulation via LMP [226,227].

EBV positivity is an emerging predictive biomarker of gastric cancer. A distinctive histological finding of gastric cancer, lymphoid stroma [228], is a molecular pathological clue of not only microsatellite instability [229] but also EBV positivity [230]. EBV-positive gastric cancer has a better prognosis than that of negative cases. Based on TCGA, the EBV subtype accounts for 9% of gastric carcinoma cases, and its unique feature is the frequent amplification of PD-L1 and PD-L2 [231]. Therefore, checkpoint inhibitor therapy is expected to be effective for EBV+GC. 

EBV-associated smooth muscle tumor (EBV+SMT) is an intermediate soft tissue neoplasm. Data for this rare entity are lacking in TCGA [232] and PCAWG [29]. The mesenchymal tumor was initially observed in clinical settings in individuals with immunosuppression. Consistent with the initial speculation [233], EBV has been detected in HIV-associated smooth muscle tumors [234]. Now, this mesenchymal tumor has been classified into three etiologies, HIV-associated, post-transplantation, and primary immunodeficient EBV+SMT [235]. The vast majority of primary immunodeficient EBV+SMT cases are pediatric multicentric lesions, whereas patients with secondary immunodeficient EBV+SMT are predominantly adults, especially after organ transplant. Rarely, EBV+SMT arises from iatrogenic immunosuppression for autoimmune disease [236]. Promising therapeutic targets are MYC overexpression and AKT/mTOR pathway activation [237,238].

### 3.4. Kaposi Sarcoma Herpes Virus (KSHV/HHV-8)

In 1994, Kaposi sarcoma herpes virus (KSHV), also known as human herpesvirus 8 (HHV-8), was discovered in a case of Kaposi sarcoma [239]. KSHV latently infects B-cells [240,241] and then expands to vascular endothelial cells [242]. The KSHV genome contains genes encoding essential components (i.e., DNA polymerase), as well as homologues, including, Bcl-2, cyclin D1, and interleukin-6 (IL-6). Of note, viral IL-6 enhances proliferation and angiogenesis in Kaposi sarcoma [243].

Latency-associated nuclear antigen-1 (LANA-1), encoded by open reading frame 73 (*ORF73*), plays an important role in the duplication and stabilization of episomal KSHV [244] in addition to the gain of oncogenic properties [245,246]. In other words, KSHV-infected cells latently proliferate under LANA-1 expression without active virus replication. 

Kaposi sarcoma (KS) is a locally aggressive vascular endothelial neoplasia, typically arising in the skin. Clinico-epidemiologically, KS is classified into four types: classic indolent KS, endemic African KS, AIDS-associated KS, and iatrogenic KS. KSHV is consistently detected in all subtypes, suggesting that the pathogenesis is associated with epidemiological distribution and/or immunosuppression [247,248]. Although clinical KS shows a multistage disease process (patch, plaque, and nodular stages), the lesions may show regression [249]. Anti-LANA antibody is used as a diagnostic marker in KS [250]. The standard therapy for KS is currently highly active anti-retroviral therapy with systemic chemotherapy in the advanced stage [251].

KSHV infection also leads to the emergence of KSHV-related hematolymphoid proliferation. These hematopoietic lesions include primary effusion lymphoma (PEL) [170,171], HHV8-positive multicentric Castleman disease (HHV8+MCD) [252], HHV8-positive diffuse large B-cell lymphoma, NOS (HHV8+DLBCL) [253], and HHV8-positive germinotropic lymphoproliferative disorder (GLPD) [254,255,256] (Table 5). 

Despite the B-cell origin, PEL expresses CD138 but lacks CD19 and CD20, suggesting that this unique terminally differentiated B-cell phenotype is a diagnostic marker for PEL in AIDS-associated lymphomas. Consistent with the findings for KS, PEL and its solid variant [257,258] always express LANA-1. Therefore, HHV8-negative common effusion lymphoma (HENCEL) should be considered a distinct entity [259]. On the other hand, HHV8+MCD is a systemic lymphoproliferative disorder with KSHV-infected B-cells, polyclonal gammaglobulinemia, and a high concentration of serum IL-6 [261]. The majority of cases arise from HHV8+MCD in patients with AIDS [262]. Notably, large B-cell lymphoma often arises in the HHV8+MCD background [253]. The tumor cells show a large plasmablastic morphology with LANA-1 expression, whereas plasmablastic lymphoma, affecting the oral cavity of patients with AIDS, are KSHV-negative [177,178]. Taken together, KSHV-related hematolymphoid lesions can be distinguishable by LANA-1 expression in B-cells. However, the treatment of these lesions is still a big clinical challenge.

### 3.5. Merkel Cell Polyoma Virus (MCV)

MCV was first isolated from Merkel cell carcinoma (MCC) [263] by the digital transcriptome subtraction method [264]. MCV has at least five viral genes, VP1, VP2, VP3, and small and large T antigen (LT). LT is a principal protein in carcinogenesis, as in other polyoma viruses [265,266]. LT functions in both Rb pathway inactivation via the Rb binding site [267,268] and viral replication by the helicase domain at the C-terminus [269]. However, MCV in MCC harbors frequent nonsense mutations in the LT gene, indicating that mutant MCV fails to replicate due to the lack of the helicase domain. In addition, MCC is associated with MCV integration with the disconnected C-terminal region of LT [270]. These LT modifications are a probable oncogenic event in Merkel cell carcinogenesis. Other recurrent genomic changes in MCC include mutations in *TP53*, *Rb,* and *PIK3CA* [271] and *L-myc* amplification [272].

MCC has several synonyms, including primary cutaneous neuroendocrine carcinoma, trabecular carcinoma, and Toker tumor [273]; however, its origin remains unclear. Before the virus was isolated, UV damage was identified as a probable cause of MCC because this rare carcinoma typically occurs in sun-damaged skin of elderly individuals. Like other virus-related tumors, MCC is also associated with immunosuppression [274]. MCC sometimes regresses spontaneously despite its highly aggressive malignant behavior [275]. Elimination may be possible by the anti-tumor immune response, as in malignant melanoma [276].

A diagnostic marker of MCC is an anti-LT antibody, CM2B4, generated from exon 2 of the LT gene in MCV [277]. Nevertheless, in a minority of MCC cases, MCV and CM2B4 expression are lacking [278]. Compared with MCV-positive MCC, MCV-negative MCC tends to harbor more frequent driver mutations mentioned above and a greater tumor mutation burden as a result of UV damage [279]. In addition, combined squamous and neuroendocrine carcinoma of the skin is MCV-negative [277,279], suggesting that MCV-negative cutaneous neuroendocrine carcinoma is a pure UV damage-driven cancer, unlike MCV-positive MCC. Another diagnostic clue is a dot-like CK20-expression pattern; however, a CK20-negative form exists [277,280].

Interestingly, tumor infiltrating lymphocytes, predictive biomarkers of immunotherapy [281,282], have recently been identified as a favorable prognostic marker of MCC [283]. Therefore, checkpoint blockade therapy is promising for the treatment of MCC [284].

### 3.6. Human T-Cell Leukemia Virus Type 1 (HTLV-1)

HTLV-1 is a deltaretrovirus that was first isolated in 1980 [285] after initial reports of adult T-cell leukemia/lymphoma (ATLL) in Japan [286]. HTLV-1 infection requires contact between the HTLV-1-infected cell and intact cell because the viral particle has a low infection efficiency [287]. By reverse transcriptase [288], HTLV-1 proviral DNA is randomly integrated into the host genome [289]. For the detection of HTLV-1, an anti-ATLL antibody (ATLA) is used owing to the scarcity of the viral particles in human sera [290]. HTLV-1 induces not only ATLL but also various HTLV-1-associated reactive lesions, including HTLV-1-associated myelopathy (HAM) [291] and HTLV-1-associated uveitis [292].

In HTLV-1-related carcinogenesis, Tax and HTLV-1 bZIP factor (HBZ) are cardinal oncogenes. The transactivator Tax induces viral replication and immortalization of human T-cells [293,294]. However, the function of Tax in ATLL is frequently suppressed by genetic and epigenetic alterations [295,296], suggesting that this gene is unnecessary in the later phase of the malignant process. In contrast, HBZ is essential for the malignant process of ATLL [297,298,299]. Interestingly, HBZ not only represses HTLV-1 transcription by CREB-2 inactivation [300] but also counteracts Tax [301,302,303], suggesting that the abovementioned Tax suppression can promote HBZ-mediated signaling.

ATLL is a CD4+ T-cell malignancy, whose atypical lymphocytes are called flower cells [304]. ATLL tissues typically express CD2, CD3, CD4, CD25, and HLA-DR, and this immunophenotype resembles that of helper/inducer T-cells. In addition, ATLL tissues frequently express a regulatory T-cell marker, FOXP3 [305]. The age of onset of ATLL is nearly 60 years, and it is classified into four clinical types, smoldering, chronic, lymphoma, and acute types [306]. The poor prognosis of ATLL, especially acute and lymphoma types, can be explained by the treatment-refractory phenotype and opportunistic infections.

CC chemokine receptor 4 (CCR4), also known as K5-5 [307], CMKBR4 [308], or CD194 [309], is a therapeutic target for HTLV-1-related diseases [310]. CCR4 expression is specific to Th2 [311] (CD4+ CCR4+) and Treg [309] (CD4+ CD25+) cells. Consistent with its counterpart, CCR4 is frequently expressed in ATLL [312,313]. Interestingly, a genomic analysis revealed that one-third of ATLL cases involve *CCR4* mutations [314]. These mutations increase CCR4 expression [315,316] and may have predictive value for the response to the anti-CCR4 therapeutic agent mogamulizumab [317]. Anti-CCR4 immunotherapy is a promising treatment for HAM [318].

### 3.7. Additional Tumor Viruses

Because MCV was first identified in 2008, there is still room for improving our understanding of tumor viruses. For example, we describe three novel candidate tumor viruses, human cytomegalovirus (CMV/HHV-5), human herpesvirus-6 (HHV-6) and adeno-associated virus-2 (AAV-2).

#### 3.7.1. Human Cytomegalovirus (CMV/HHV-5)

The largest human herpesvirus, CMV/HHV-5, was firstly isolated in 1957 from an infant with cytomegalic inclusion disease [319], following the isolation of mouse cytomegalovirus (also called as salivary gland virus) in 1954 [320]. The owl’s eye appearance of inclusion bodies is a well-established histological clue of CMV infected cells [321]. Like other herpesviruses, CMV latently infects most individuals during childhood. However, the viral proliferation causes organopathy in immunodeficient individuals, including fetuses or immunocompromised hosts such as post-transplant and AIDS patients [322,323]. To prevent the life-threatening disease, detection of CMV antigenemia using with the tegument protein pp65 (UL83) [324,325] and prophylactic and preemptive therapy [326] are performed in clinical setting.

Although latent CMV infection itself is believed to be asymptomatic and harmless under normal immune status, its enigmatic oncogenic functions in breast cancer have been attracting attention [327]. “Oncomodulation”, proposed by Michaelis et al. [328], is defined as enhanced malignant progression of tumor cells through CMV infection. Like survival in the infected normal cells, CMV products affect various tumor cell properties, cell cycle, apoptosis, metastasis, angiogenesis, and immune evasion. In addition, a specific CMV strain has been reported to transform human mammary epithelial cells into triple-negative breast cancer-like cells [329]. Consistent with these findings, the IARC advisory board group recently recommended CMV as a high priority agent to be evaluated for its carcinogenicity [12].

#### 3.7.2. Human Herpesvirus-6 (HHV-6)

HHV-6 was initially named human B-lymphotropic virus [330] but was subsequently identified as a herpesvirus [331]. HHV-6 consists of two distinctive variants, HHV-6A and HHV-6B, based on biological, clinical and epidemiological differences, despite the overall highly conserved genetic sequences [332,333]. The latter is a cause of exanthema subitem [331] and HHV-6B encephalitis [334] whereas the former still remains unclear with respect to initial clinical symptoms. Except for infants, almost all individuals have latent HHV-6 infection [335,336]. HHV-6 latently infects bone marrow progenitor cells [337] and is integrated into the human genome [338]. Notably, this integration includes the whole genome of HHV-6 and also occurs in germ cells [339,340]. Similarly, tumor tissues sometimes contain HHV-6, although a direct oncogenic effect of HHV-6 has not yet been demonstrated [341]. However, accumulating evidence suggests that an HHV-6 viral protein, direct repeat 7 (DR7), may function as an oncoprotein through the p53 downregulation in EBV-negative lymphoma and glioma [342,343,344]. To clarify the molecular significance of HHV-6, further investigations are needed.

#### 3.7.3. Adeno-Associated Virus-2 (AAV-2)

AAV is a minute parvovirus, discovered as a small particle in the preparation of adenovirus [345,346]. Owing to the unique biological properties of AAV-2 [347], AAV is utilized as a gene therapy vehicle [348]. Currently, retroviruses and AAV-2 are potent vectors to induce external gene expression. Although retroviruses enable persistent gene expression by genomic integration of the viral sequence, the alteration can lead to insertional mutagenesis [349,350,351]. In contrast, AAV-2 is believed to be safe owing to its extremely low integration rate, except in a single fatal clinical report [352]. However, a long-term observational study revealed that AAV-treated mice harbor neoplastic cells in the liver [353,354]. The AAV sequence fragment has been detected in HCC, like HBV-related mutagenesis [355]. AAV administration results in the induction of hepatic proliferative lesions in dogs [356]. However, contrary results have been reported [357,358]. In fact, the liver is one of most frequent AAV detection sites in humans [359]. Taken together, the relationship between AAV and hepatocellular carcinogenesis is still controversial.

Further studies of these novel tumor virus candidates are required.

## 4. Conclusions

In this review we described the current understanding of virus-driven carcinogenesis. Tumor viruses were overlooked for a long time, because of their invisibility and long latency. However, their oncogenic activities have been detected by our technologies. Furthermore, the nature of novel tumor virus candidates, including CMV, HHV-6, and AAV-2, is becoming unraveled. To win the war against virus-associated cancer, comprehensive approaches for cancer prevention, diagnosis, and treatment are necessary in the future.

## Figures and Tables

**Table 1:** Summary of human tumor viruses.

Variable	HPV	HBV	HCV	EBV	KSHV	MCV	HTLV-1
Full Name/ synonym	Human Papilloma Virus	Hepatitis B Virus	Hepatitis C Virus	Epstein–Barr Virus/ HHV-4	Kaposi Sarcoma Herpes Virus/HHV-8	Merkel Cell Polyoma Virus	Human T-cell Lymphotrophic Virus, type-1
Family	*Papillomaviridae*	*Hepadnaviridae*	*Flaviviridae*	*Herpesviridae*	*Herpesviridae*	*Polyomaviridae*	*Retroviridae*
Baltimore System ^1^	Class I	Class VII	Class IV	Class I	Class I	Class I	Class VI
Virus Genome	dsDNA	ss/dsDNA	+ssRNA	lineardsDNA	circulardsDNA	dsDNA	+RNA
Genome Size	8 kbp	3 kb/kbp	9.5–12.5 kb	170 kbp	170 kbp	5.4 kbp	9 kbp
Virion Size	52–60 nm	52–55 nm	40–60 nm	200 nm	100–150 nm	40–55 nm	100 nm
Envelope	Absent	Present	Present	Present	Present	Absent	Present
Capsid	Icosahedral	Icosahedral	Icosahedral	Icosahedral	Icosahedral	Icosahedral	Icosahedral
Vaccination	Accessible	Accessible	Inaccessible	Inaccessible	Inaccessible	Inaccessible	Inaccessible
Anti-viral treatment	Not established	Effective	Effective	Occasionally Effective	Not established	Not established	Not established
Diagnostic molecule	p16	HBs antigen	anti-HCV antibody	EBER	LANA	CM2B4	ATLA

^1^ The details of Baltimore system are found in refs. [13,14]. Abbreviations: ATLA, anti-adult T-cell leukemia/lymphoma antibody; ds, double strands; EBER, EBV-encoded small RNA; LANA, Latency-associated nuclear antigen; ss, single strand; +, positive sense.

**Table 2:** Summary of virus-driven carcinogenesis.

Virus	Target Organ	Tumor Type	Common Features
High-risk HPVs	Uterine cervix	Squamous cell carcinoma	p16 positive, favorable prognosis
		Adenocarcinoma	
	Head and neck (oropharynx)	Squamous cell carcinoma	
	Vagina	Squamous cell carcinoma	
	Vulva	Squamous cell carcinoma	
	Penis	Squamous cell carcinoma	
HBV	Liver	Hepatocellular carcinoma	Frequent *TP53* mutation
		Cholangiocellular carcinoma	Small duct type
HCV	Liver	Hepatocellular carcinoma	Frequent *TERT* mutation
		Cholangiocellular carcinoma	Small duct type
	Hematopoietic system	Malignant lymphoma	Possible regression by viral elimination
EBV/ HHV-4	Stomach	Adenocarcinoma	EBER positive, frequent TILs
	Nasopharynx	Nasopharyngeal carcinoma	EBER positive, frequent TILs
	Hematopoietic system	See Tables 3 and 4	EBER positive
	Soft tissue	EBV-associated smooth muscle tumor	EBER positive
KSHV/ HHV-8	Soft tissue	Kaposi sarcoma	LANA positive
	Hematopoietic system	See Table 5	
MCV	Skin	Merkel cell carcinoma	CM2B4 positive
HTLV-1	Hematopoietic system	Adult T-cell leukemia/lymphoma	Frequent *CCR4* mutation

Abbreviations: EBER, EBV-encoded small RNA; LANA, Latency-associated nuclear antigen; TILs, Tumor infiltrating lymphocytes.

**Table 3:** The latency patterns of EBV-associated cancers.

Variable	Latency I	Latency II	Latency III
EBER	Positive	Positive	Positive
EBNA1	Positive	Positive	Positive
EBNA2	Negative	Negative	Positive
LMP1	Negative	Positive	Positive
Histological type	Burkitt lymphoma EBV+ gastric cancer	CHL (50%) EBV+DLBCL (common) CAEBV/HVLPD/SMBA PEL ENKTL NPC AITL OI-LPD (common)	EBV+SMT EBV+DLBCL (uncommon) LYG PAL/FA-DLBCL PTLD OI-LPD (uncommon)

Abbreviations: AITL, Angioimmunoblastic T-cell lymphoma; CAEBV, Chronic active HBV infection of T- and NK-cell type, systemic form; CHL, Classical Hodgkin lymphoma; EBER, EBV-encoded small RNA; EBNA, EBV-encoded nuclear antigen; EBV+DLBCL, EBV-positive diffuse large cell B-cell lymphoma, not otherwise specified (NOS); EBV+SMT, EBV-associated smooth muscle tumor; ENKTL, Extranodal NK/T-cell lymphoma, nasal type; FA-DLBCL, Fibrin-associated diffuse large cell B-cell lymphoma; HVLPD, Hydroa vacciniforme-like lymphoproliferative disorders; LMP, Late membrane protein; LYG, Lymphomatoid granulomatosis; NPC, Nasopharyngeal carcinoma; OI-LPD, Other iatrogenic lymphoproliferative disorder; PAL, Pyotholax-associated lymphoma; PEL, Primary effusion lymphoma; SMBA, severe mosquito bite allergy; PTLD, Post-transplant lymphoproliferative disorders.

**Table 4:** Association between hematolymphoid lesions and EBV.

Category	Diagnostic Entity (Abbreviation)	Clonality	Common Features	References	Virus Negative Mimicry	References
B	Burkitt lymphoma	Monoclonal	*MYC* translocation, Ki-67 99%	[135,147,153,154,155,156]	Burkitt-like lymphoma with 11q aberration	[158,159]
B	EBV-positive diffuse large B-cell lymphoma, not otherwise specified (NOS) (EBV+DLBCL)	Monoclonal	B-cell markers (CD19, CD20, CD22, CD79)+, MUM1+, CD10−	[161,162,163]	Diffuse large B-cell lymphoma, NOS (DLBCL)	[164]
B	EBV-positive mucocutaneous ulcer (EBVMCU)	Variable	Immunosuppression, methotrexate	[165]	Diffuse large B-cell lymphoma, NOS (DLBCL)	[164]
B	Pyotholax-associated lymphoma (PAL)	Monoclonal	PAX5+, *TP53* mutation, *MYC* amplification	[166,167,168,169]	Primary effusion lymphoma (PEL)	[170,171]
B	Fibrin-associated diffuse large B-cell lymphoma (FA-DLBCL)	Monoclonal	CD30+, MUM1+, PD-L1+, intact *MYC*	[172,173]	Primary effusion lymphoma (PEL)	[170,171]
B	Lymphomatoid granulomatosis (LYG)	Variable	CD20+, CD30+/−, CD15−	[174,175]	Granulomatosis with polyangiitis (Wegener’s)	[176]
B	Plasmablastic lymphoma	Monoclonal	EBER+/−, *MYC* translocation	[177,178]	Plasmablastic plasma cell myeloma	[179]
B	Primary effusion lymphoma (PEL)	Monoclonal	EBER+/−, *MYC* translocation	[170,171]	EBV-negative PEL	[170,171]
NK/T	Aggressive NK-cell leukemia (ANKL)	Monoclonal	CD2+, surface CD3−, CD3-epsilon+, CD5−, CD56+	[180,181]	EBV-negative aggressive NK-cell leukemia	[182]
NK/T	Systemic EBV+T-cell lymphoma of childhood	Monoclonal	CD8+, CD2+, surface CD3+, TIA1+, granzyme B+	[183,184]	Non EBV-associated hemophagocytic syndrome	[185]
NK/T	Chronic active EBV infection of T-and NK-cell type, systemic form (CAEBV)	Monoclonal	TIA1+, granzyme B+	[186,187]	Inflammation of unknown etiology	[188]
NK/T	Hydroa vacciniforme-like lymphoproliferative disorders (HVLPD)	Monoclonal	CD3+, CD56−, TIA1+, granzyme B+	[189,190]	Hydroa vacciniforme	[191]
NK/T	Severe mosquito bite allergy (SMBA)	Monoclonal	CD3-epsilon+, CD56+	[192,193]	Not applicable	
NK/T	Extranodal NK/T-cell lymphoma, nasal type (ENKTL)	Monoclonal	CD3+, CD56+, *GNAQ* mutation	[194,195]	Other NT/T-cell lymphomas	[196]
NK/T	Angioimmunoblastic T-cell lymphoma (AITL)	Monoclonal	EBER−, EBV+ B-cell, *RHOA* and *TET2* mutation	[197,198]	Peripheral T-cell lymphoma, NOS (PTCL)	[199]
NK/T	Follicular T-cell lymphoma (FTCL)	Monoclonal	EBER−, EBV+ H/RS-like cells, *ITK-STK* fusion	[200,201,202]	Peripheral T-cell lymphoma, NOS (PTCL)	[203]
NK/T	Nodal peripheral T-cell lymphoma with T follicular helper phenotype	Monoclonal	Possibility of tumor cell rich variants of AITL	[204,205]	Peripheral T-cell lymphoma, NOS (PTCL)	[206]
Hodgkin	Classic Hodgkin lymphoma (CHL)	Monoclonal	CD30+, CD15+, Mixed cellularity	[207,208]	Nodular lymphocyte predominant Hodgkin lymphoma (NPDHL)	[209]
IA-LPD	Lymphoproliferative diseases associated with primary immune disorders (PID)	Variable	Various lesions and etiologies	[152,210]	Various lesions	[152]
IA-LPD	Lymphomas associated with HIV infection	Monoclonal	Various lesions, including, PEL, plasmablastic lymphoma and HHV8+DLBCL	[211,212]	Not applicable	
IA-LPD	Post-transplant lymphoproliferative disorders (PTLD)	Variable	EBV+ (most cases)	[152]	EBV-negative PTLD	[213,214]
IA-LPD	Other iatrogenic immunodeficiency-associated lymphoproliferative disorders (OI-LPD)	Variable	Immunosuppressive drugs, including methotrexate	[215]	Various lesions	[152]

Abbreviations: EBER, EBV-encoded small RNA; EBV+ H/RS-like cells, EBV-positive Hodgkin/Reed–Sternberg-like cells; HHV8+DLBCL, HHV8-positive diffuse large B-cell lymphoma, NOS; IA-LPD, Immunodeficiency associated-lymphoproliferative disorder.

**Table 5:** Association between hematolymphoid lesions and KSHV.

Cell Linage	Diagnostic Entity (Abbreviation)	Clonality	Common Features	References	Virus Negative Mimicry	References
B	Primary effusion lymphoma (PEL) and its solid variants	Monoclonal	CD20-, HIV+/−, EBV+/−	[170,171] [257,258]	HHV8 negative common effusion lymphoma (HENCEL)	[259]
B	HHV8-positive multicentric Castleman disease (HHV8+MCD)	Polyclonal	MUM1+, CD138−, HIV+/−, EBV−	[252]	HHV8-negative multicentric Castleman disease (HHV8-MCD)	[260]
B	HHV8-positive diffuse large B-cell lymphoma, NOS (HHV8+DLBCL)	Monoclonal	CD20+/−, CD79a-, CD138−, HIV+/−, EBV−	[253]	Plasmablastic lymphoma	[177,178]
B	HHV8-positive germinotropic lymphoproliferative disorder (GLPD)	Polyclonal/ oligoclonal	MUM1+, CD138−, HIV−, EBV+	[254,255,256]	Plasmablastic lymphoma	[177,178]
